# High plasma coenzyme Q10 concentration is correlated with good left ventricular performance after primary angioplasty in patients with acute myocardial infarction

**DOI:** 10.1097/MD.0000000000004501

**Published:** 2016-08-07

**Authors:** Ching-Hui Huang, Chen-Ling Kuo, Ching-Shan Huang, Wan-Min Tseng, Ie Bin Lian, Chia-Chu Chang, Chin-San Liu

**Affiliations:** a Division of Cardiology, Department of Internal Medicine, Changhua Christian Hospital; b Institute of Statistics and Information Science, National Changhua University of Education; c Vascular and Genomic Research Center; d Division of Nephrology, Department of Internal Medicine, Changhua Christian Hospital, Changhua; e School of Medicine, Chung Shan Medical University, Taichung; f Department of Neurology, Changhua Christian Hospital, Changhua; g Graduate Institute of Integrative Medicine, China Medical University, Taichung, Taiwan.

**Keywords:** acute ST segment elevation myocardial infarction, left ventricular systolic function, plasma coenzyme Q10

## Abstract

Exogenous administration of coenzyme Q10 (CoQ10) has been shown in experimental models to have a protective effect against ischemia–reperfusion injury. However, it is unclear whether follow-up plasma CoQ10 concentration is prognostic of left ventricular (LV) performance after primary balloon angioplasty in patients with acute ST segment elevation myocardial infarction (STEMI).

We prospectively recruited 55 patients with STEMI who were treated with primary coronary balloon angioplasty. Plasma CoQ10 concentrations were measured before primary angioplasty (baseline) and 3 days, 7 days, and 1 month after STEMI using high-performance liquid chromatography. Echocardiography was performed at baseline and at 6-month follow-up. The control group comprised 54 healthy age- and sex-matched volunteers.

Serial circulating CoQ10 concentrations significantly decreased with time in the STEMI group. The LV ejection fraction at 6-month follow-up positively correlated with the 1-month plasma CoQ10 tertile. Higher plasma CoQ10 concentrations at 1 month were associated with favorable LV remodeling and systolic function 6 months after STEMI. Multiple linear regression analysis showed that changes in CoQ10 concentrations at 1-month follow-up were predictive of LV systolic function 6 months after STEMI. Changes in CoQ10 concentrations correlated negatively with baseline oxidized low-density lipoprotein and fibrinogen concentrations and correlated positively with leukocyte mitochondrial copy number at baseline.

Patients with STEMI who had higher plasma CoQ10 concentrations 1 month after primary angioplasty had better LV performance at 6-month follow-up. In addition, higher plasma CoQ10 concentration was associated with lower grade inflammatory and oxidative stress status. Therefore, plasma CoQ10 concentration may serve as a novel prognostic biomarker of LV systolic function after revascularization therapy for acute myocardial infarction.

## 1 Introduction

Although reestablishment of blood flow after myocardial infarction via percutaneous coronary intervention (PCI) or other interventions is effective in the short term, long-term functional recovery is not guaranteed, thereby putting patients at risk for further cardiac events.^[1–4]^ Recovery of myocardial function after revascularization occurs gradually over a period of several months because it is dependent on the recovery of mitochondrial function.^[5]^ Holley et al postulated that normalization of oxidative phosphorylation by the mitochondrial electron transport chain may be required for complete myocardial recovery.^[5]^

Coenzyme Q10 (CoQ10), an endogenous antioxidant localized to the mitochondrial membrane that facilitates electron transport, plays a key role in oxidative phosphorylation.^[6]^ Pretreatment with or infusion of CoQ10 soon after coronary artery ligation has been shown to reduce infarct size and preserve systolic function in rat models of acute myocardial infarction (AMI).^[7,8]^ Accumulation of CoQ10 within mitochondria has also been shown to improve mitochondrial function.^[9]^ In addition, CoQ10 is a powerful free radical scavenger that can mitigate DNA damage caused by oxidative stress.^[6]^ Mitochondrial DNA (mtDNA) is situated near the inner mitochondrial membrane and electron transport system, making it prone to oxidative stress from the electron transport system.^[10,11]^ Therefore, any damage to mtDNA potentially induces dysfunctional mitochondrial transcripts and reduces oxidative phosphorylation.^[12]^ Evidence suggests that mtDNA damage and defects in electron transport function play an important role in the development and progression of cardiac remodeling and failure.^[13]^ Expression of mtDNA-encoded genes is largely regulated by the mtDNA copy number (MCN) and maintenance of the MCN is important for the preservation of mitochondrial function.^[14]^

The aims of this study were to evaluate whether endogenous plasma CoQ10 concentration is predictive of left ventricular (LV) performance after primary angioplasty in patients with ST segment elevation myocardial infarction (STEMI) and to elucidate the relationship between CoQ10 concentration and mitochondrial function in patients with STEMI.

## 2 Subjects and methods

### 2.1 Subjects and study protocol

In this prospective study, we enrolled 55 consecutive patients with de novo acute STEMI who underwent primary PCI and thromboaspiration during the period January 2010 to January 2011 at the Changhua Christian hospital, a 1500-bed tertiary hospital in Taiwan. The mean door-to-balloon time was 84.9 ± 38.0 minutes. All eligible patients aged 18 to 80 years who consented to participate in the study were included (n = 55). Venous blood was obtained prior to PCI and 3 days, 7 days, and 1 month after the acute event. Plasma was collected by centrifugation for 10 minutes at 1000 × *g* and 4°C, divided into several aliquots, and stored at −80°C until analysis. Baseline creatine phosphokinase and plasma CoQ10 concentrations were routinely measured prior to PCI. Baseline lipid and glucose concentrations were measured after an 8-hour fast. The creatine kinase-MB fraction and troponin I concentrations were measured every 4 hours until they started to decline and the peak values were recorded. Diagnosis of STEMI was primarily based on the Joint Task Force for the Universal Definition of Myocardial Infarction as reported by Thygesen et al and in our previous study.^[15,16]^ Briefly, the diagnostic criteria used in this study included ST segment elevation of 0.2 mV in ≥2 contiguous electrocardiography leads and an increase in cardiac biomarkers (e.g., troponin I and creatinine kinase-MB fraction) with at least 1 value above the 99th percentile of the upper reference limit within 24 hours of the onset of pain. The culprit vessel was identified based on clinical, electrocardiographic, and angiographic findings. All patients were placed on 100 mg of aspirin and 300 mg of clopidogrel prior to PCI. After PCI, patients received standard care including administration of aspirin, clopidogrel, β-blockers, statins, angiotensin-converting enzyme inhibitors, or angiotensin receptor blockers as appropriate. Patients also received smoking cessation and lifestyle counseling. None of the subjects took vitamin or CoQ10 supplements before STEMI and all were instructed not to change their dietary habits during the study period. Echocardiographic examinations were performed within the first 2 days after primary PCI and 6 months later. The echocardiographic images were acquired by the same qualified cardiologist, who was blinded to the patients’ plasma CoQ10 concentrations. Baseline concentrations of plasma CoQ10 and apolipoprotein A1 (ApoA1) in the 55 patients with STEMI were compared with those in 54 healthy age- and sex-matched volunteers. Only patients in the STEMI group underwent serial venous blood follow-up. The protocol was approved by the Institutional Review Board of the Changhua Christian Hospital, Taiwan, and all subjects gave written informed consent to participate.

### 2.2 Measurement of plasma biochemical parameters

#### 2.2.1 Plasma CoQ10 measurement

The plasma concentration of CoQ10 was determined as described in our previous study.^[17,18]^ Briefly, frozen plasma samples were thawed on ice and then maintained at 4°C during handling. Thawed plasma was pipetted into Eppendorf microcentrifuge tubes, deproteinized with methanol, and then treated with *n*-hexane. The mixture was vortexed for 5 minutes and then centrifuged at 2500 × *g* and 4°C for 15 minutes, after which the clear *n*-hexane layer was transferred to another tube for another round of *n*-hexane extraction. The plasma extracts were combined and evaporated to dryness under a gentle stream of nitrogen gas. The dry residue was dissolved in the mobile phase, filtered through a membrane (polyvinylidene fluoride filter, 4 mm, 0.45 μm; Millipore, Bedford, MA), and injected into a high-performance liquid chromatography system. A microBondapak C18 3.9-mm × 30.0-cm stainless steel column with a 3 × 22–mm guard column packed with microBondapak C18 was used with the mobile phase of methanol–*n*-hexane 85:15 (v/v). The flow rate was 1 mL/min. The wavelength of the ultraviolet detector was fixed at 276 nm. To determine the amount of CoQ10 in plasma, a calibration curve was constructed by plotting the peak areas versus the concentration. Linearity was achieved in the concentration range of 0.12 to 1.92 μg/mL.

#### 2.2.2 Plasma lipid profile measurement

The plasma ApoA1 concentration was determined by an immunoturbidimetric assay according to the manufacturers’ instructions (ADVIA Chemistry System; Siemens, New York, NY). Plasma total cholesterol, high-density lipoprotein cholesterol, low-density lipoprotein cholesterol, and triglyceride concentrations were determined using a Beckman UniCel DxC 800 chemistry analyzer (Beckman Coulter Inc, Brea, CA).

#### 2.2.3 Determination of the MCN in human leukocytes

The procedure for determining MCNs in leukocytes was performed as described in our previous studies.^[19,20]^ Briefly, total leukocyte DNA from the buffy coat was extracted using the Gentra Puregene DNA kit (Qiagen, Hilden, Germany) with care taken to avoid platelet contamination. Polymerase chain reaction was performed by amplification of the *ND1* gene in mtDNA and the β-globin gene in nuclear DNA from sampled DNA. The MCN in leukocytes was assessed using a LightCycler Instrument (Roche, Mannheim, Germany) and calculation of the MCN was interpolated from the linearity of a standard curve of serially diluted plasmid containing cDNA encoding the *ND1*/β-globin gene.

### 2.3 Echocardiographic assessments

All 2-dimensional echocardiographic parameters were measured according to the guidelines of the American Society of Echocardiography.^[21]^ We used a modified Simpson's rule formula to calculate the LV ejection fraction (LVEF) as described by the American Society of Echocardiography.^[22]^ Echocardiography was performed at baseline (within 2 days after PCI) and at 6-month follow-up.

### 2.4 Statistical analysis

Values are presented as mean ± standard deviation. Differences in values between the AMI group and the control group were evaluated by the Student *t* test. We used generalized estimating equations to assess the differences in plasma CoQ10 profiles over time. Generalized estimating equation is a regression technique suitable for repeated measurements and longitudinal data analysis. Receiver operating characteristic curves were constructed and the area under the curves (AUC) was calculated to assess the sensitivity and specificity of CoQ10 concentration at 1 month for predicting LVEF and remodeling at 6 months. The Jonckheere–Terpstra test for trend was used to analyze the association between CoQ10 concentration at 1 month, after the values had been divided into tertile groups, and LVEF at 6-month follow-up. The Jonckheere–Terpstra test was also used to analyze the association between baseline plasma CoQ10 concentration and baseline ApoA1 concentration. The Jonckheere–Terpstra test is similar to the Kruskal–Wallis test but is applied to samples with a priori ordering. A multivariate linear regression model was used to evaluate independent associations between possible predictors and LVEF at 6 months. Factors associated with changes in plasma CoQ10 concentration 1 month after AMI were also evaluated with the multivariate linear regression model. A *P* value of <0.05 was considered to indicate statistical significance. All statistical analyses were performed using the statistical package SPSS for Windows (version 15.0, SPSS, Chicago, IL). Because this was a pilot study, we had no reference data on plasma CoQ10 concentrations in patients with STEMI. Therefore, we were not able to estimate the sample size and study power.

## 3 Results

### 3.1 Patient characteristics in the AMI group

The baseline characteristics of the 55 patients in the AMI group and the 54 age- and sex-matched healthy volunteers are summarized in Table 1. Baseline plasma CoQ10 and ApoA1 concentrations were compared between the STEMI group and the healthy control group.

**Table 1:**
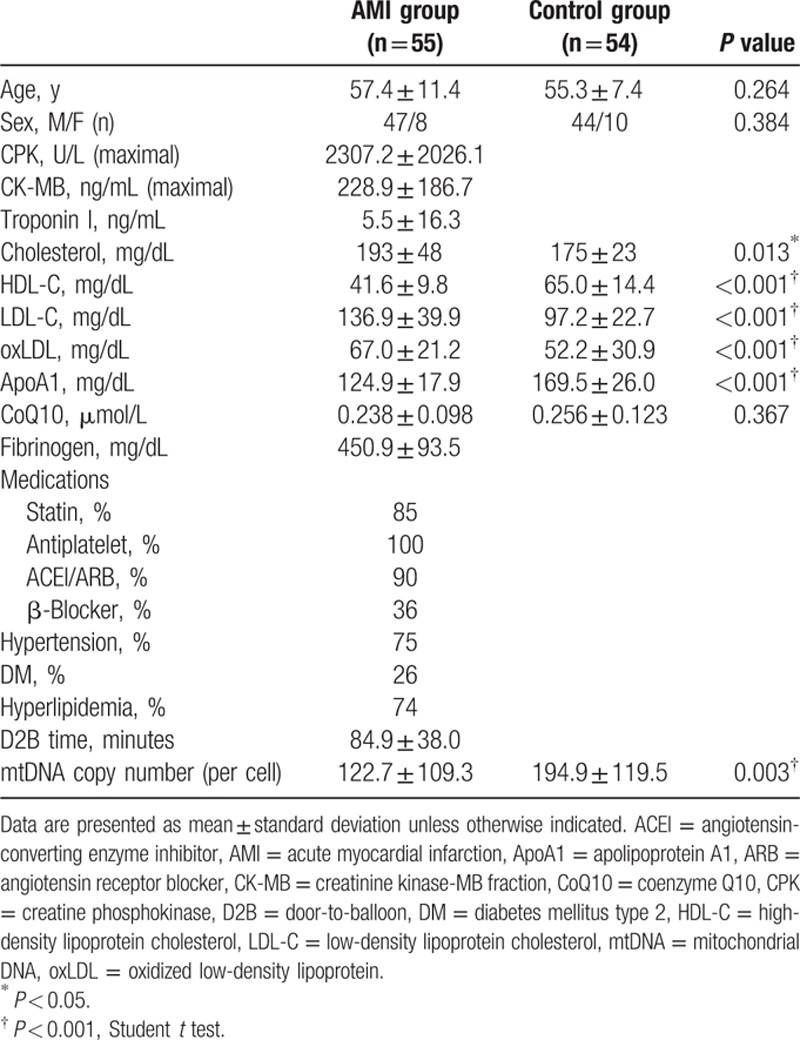
Clinical characteristics and biochemical variables of the control and AMI groups.

### 3.2 Serial circulating coenzyme Q10 levels gradually decreased with time in patients with STEMI

**Figure 1:**
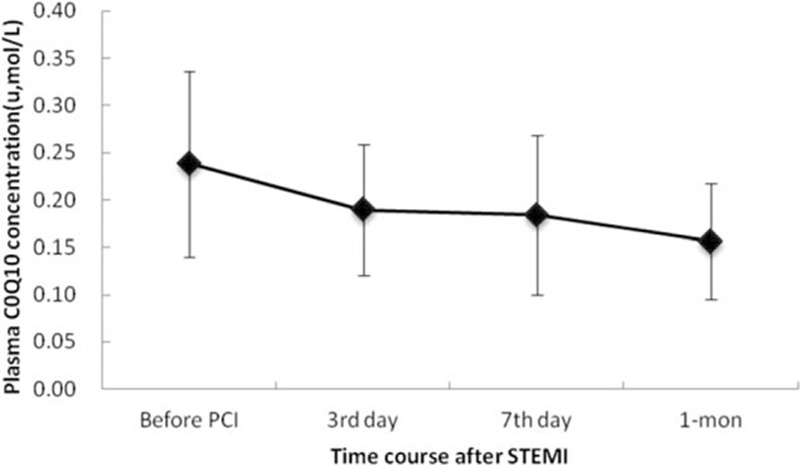
Temporal changes in plasma CoQ10 concentration after primary angioplasty in patients with STEMI. CoQ10 = coenzyme Q10, PCI = percutaneous coronary intervention, STEMI = ST segment elevation myocardial infarction.

There were no significant differences in plasma CoQ10 concentrations between the AMI group before PCI and the control group (0.238 ± 0.098 vs 0.256 ± 0.123 μmol/L, respectively; *P* = 0.367). The serial circulating CoQ10 concentrations in patients who underwent AMI decreased significantly with time (Fig. 1). The mean plasma CoQ10 concentration was 0.238 ± 0.098 μmol/L before PCI, 0.189 ± 0.068 μmol/L on day 3, 0.183 ± 0.084 μmol/L on day 7, and 0.156 ± 0.061 μmol/L at 1 month after STEMI. After adjusting for age and sex, the results of the generalized estimating equation test revealed significant differences in plasma CoQ10 levels among the 4 time points. The adjusted regression coefficients decreased significantly over time (Table 2), indicating that plasma CoQ10 levels gradually decrease after PCI.

**Table 2:**
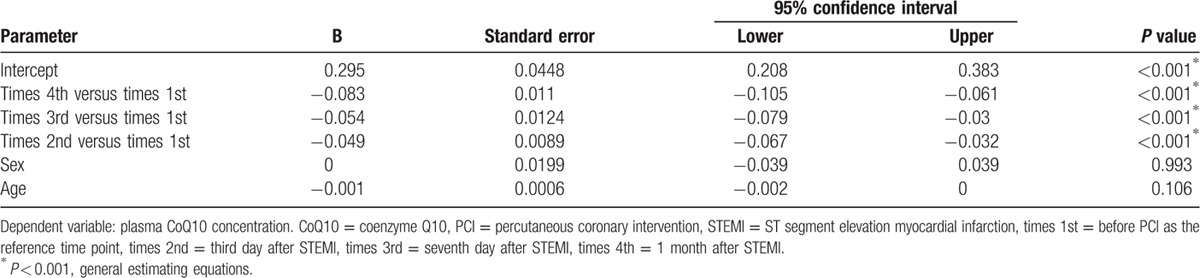
Generalized estimating equation results for plasma CoQ10 change after STEMI.

### 3.3 LVEF at 6 months was positively proportional to plasma CoQ10 concentration at 1 month

**Figure 2:**
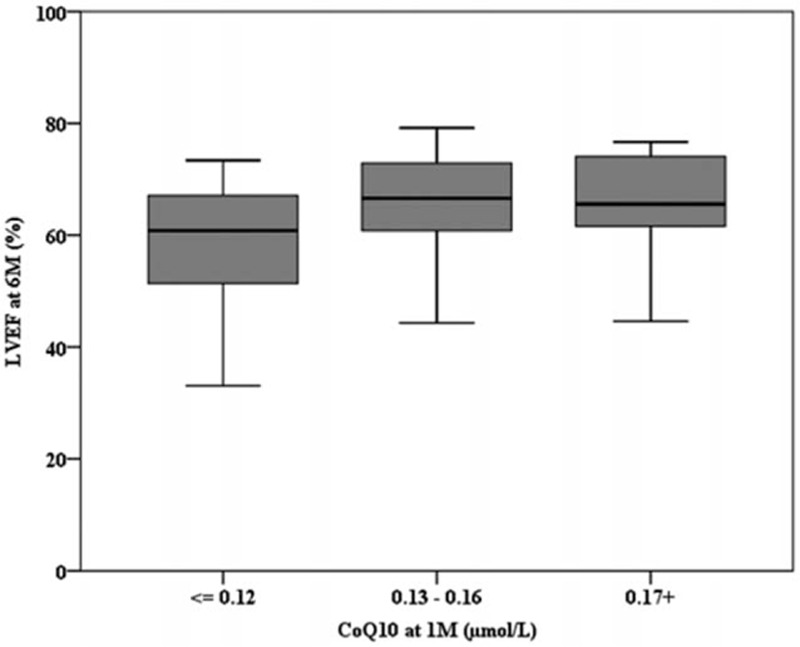
Trend analysis shows that the 6-month LVEF was positively proportional to the 1-month plasma CoQ10 concentration tertile. Patients with AMI were divided into 3 subgroups according to the 1-month plasma CoQ10 concentration tertile: Group 1, CoQ10 concentration of ≤0.12 μmol/L (n = 19); Group 2, CoQ10 concentration of 0.13 to 0.16 μmol/L (n = 20); and Group 3, CoQ10 concentration of ≥0.17 μmol/L (n = 16). Trend analysis showed that the 6-month LVEF was positively proportional to the 1-month plasma CoQ10 concentration (Jonckheere–Terpstra test, *P* = 0.026). AMI = acute myocardial infarction, CoQ10 = coenzyme Q10, LVEF = left ventricular ejection fraction.

Patients with STEMI were divided into 3 subgroups according to the 1-month plasma CoQ10 concentration tertile. Trend analysis showed that the 6-month LVEF was positively proportional to the 1-month plasma CoQ10 concentration (Jonckheere–Terpstra test, *P* = 0.026) (Fig. 2).

### 3.4 Evaluation of plasma CoQ10 concentration at 1 month as a predictor of improvement in LVEF at 6 months

**Figure 3:**
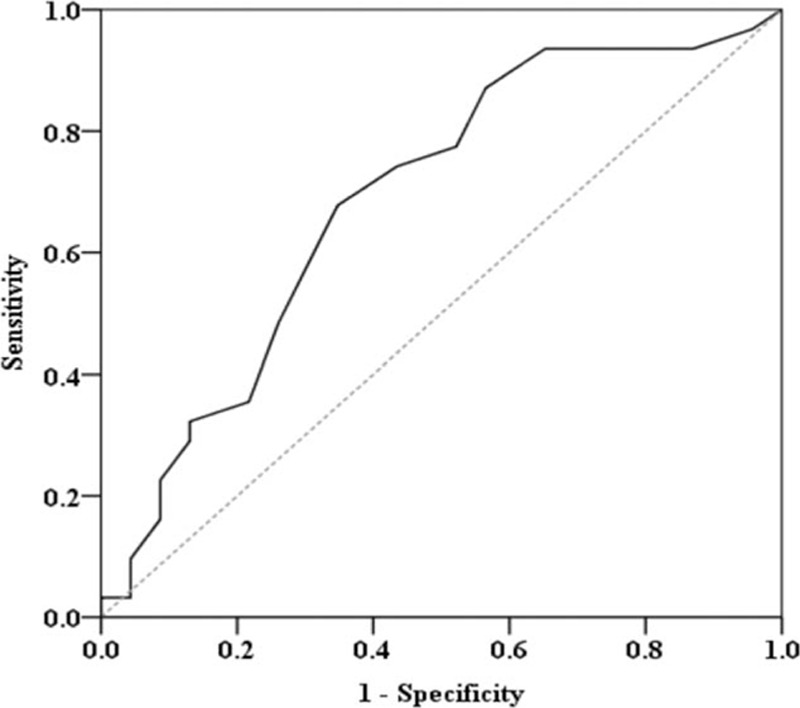
Evaluation of plasma CoQ10 concentration at 1 month as a predictor of improvement in LVEF at 6 months. The AUC for plasma 1-month CoQ10 concentration as a predictor of 6-month LVEF improvement was 0.687 (95% CI, 0.540–0.834, *P* = 0.02). AUC = area under the curves, CI = confidence interval, CoQ10 = coenzyme Q10, LVEF = left ventricular ejection fraction.

Change in heart function was calculated by subtracting the LVEF at baseline from the ejection fraction at 6 months. Improvement in heart function was defined as a change in LVEF of ≥0 at 6 months. Using this as a reference value, patients with STEMI were divided into 1 of 2 subgroups, namely, an improvement group or a nonimprovement group. The AUC for plasma CoQ10 concentration at 1 month versus LVEF improvement at 6 months was 0.687 (95% confidence interval (CI), 0.540–0.834, *P* = 0.02) (Fig. 3). At a cutoff point of 0.145 μmol/L, plasma CoQ10 concentration at 1 month had a sensitivity of 0.677 and a specificity of 0.652 for predicting LVEF at 6 months.

### 3.5 Evaluation of plasma CoQ10 concentration at 1 month as a predictor of LV remodeling at 6 months

**Figure 4:**
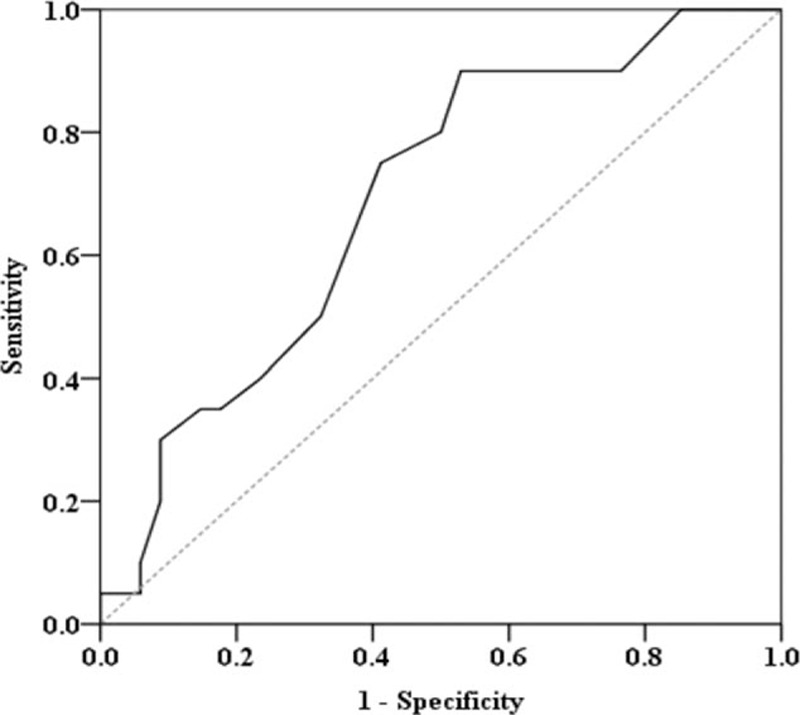
Evaluation of plasma CoQ10 concentration at 1 month as a predictor of LV remodeling at 6 months. The AUC for plasma 1-month CoQ10 concentration as a predictor of 6-month LV remodeling was 0.690 (95% CI, 0.547–0.832, *P* = 0.02). AUC = area under the curves, CI = confidence interval, CoQ10 = coenzyme Q10, LV = left ventricular.

Change in left ventricular end-systolic volume (LVESV) was calculated by subtracting the LVESV at baseline from the LVESV at 6 months and then dividing the difference by the baseline LVESV. Unfavorable LV remodeling was defined as a change in LVESV of ≥10% at 6 months. Patients with STEMI were divided into 1 of 2 subgroups, namely, a favorable LV remodeling group or an unfavorable LV remodeling group. The AUC for plasma CoQ10 concentration at 1 month versus LV remodeling at 6 months was 0.690 (95% confidence interval, 0.547–0.832, *P* = 0.02) (Fig. 4). At a cutoff point of 0.145 μmol/L, plasma CoQ10 concentration at 1 month had a sensitivity of 0.750 and a specificity of 0.588 for predicting LV remodeling at 6 months.

### 3.6 Higher CoQ10 concentration in plasma at 1 month is predictive of better LV performance 6 months after primary angioplasty for STEMI

The change in the plasma CoQ10 concentration (ΔCoQ10) was calculated by subtracting the 1-month plasma CoQ10 concentration from the baseline CoQ10 concentration. A higher ΔCoQ10 value indicated greater retention of CoQ10 in plasma at 1 month after an AMI event. Multivariate analysis revealed that the 6-month LVEF was independently associated with the ΔCoQ10 and negatively associated with infarct size as measured by peak creatinine kinase-MB mass concentration (Table 3).

**Table 3:**
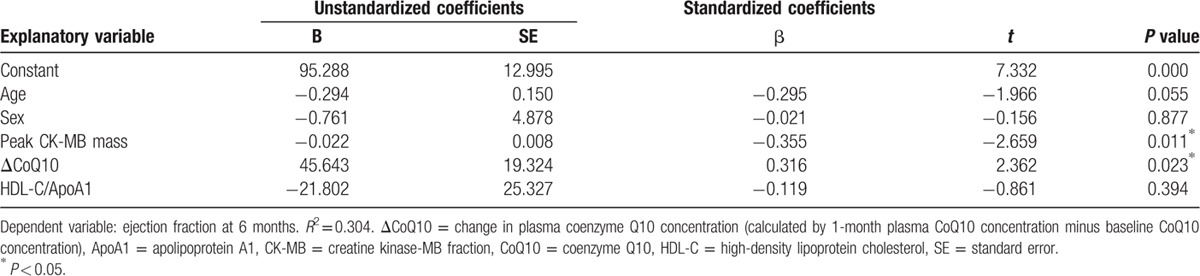
Multiple linear regression analysis of variables associated with ejection fraction 6 months after primary angioplasty for acute myocardial infarction.

### 3.7 Factors associated with retention of CoQ10 in plasma 1 month after AMI

Multivariate analyses revealed that the ΔCoQ10 was negatively associated with baseline oxidized low-density lipoprotein (oxLDL) and fibrinogen concentrations and positively associated with baseline leukocyte MCN (Table 4).

**Table 4:**
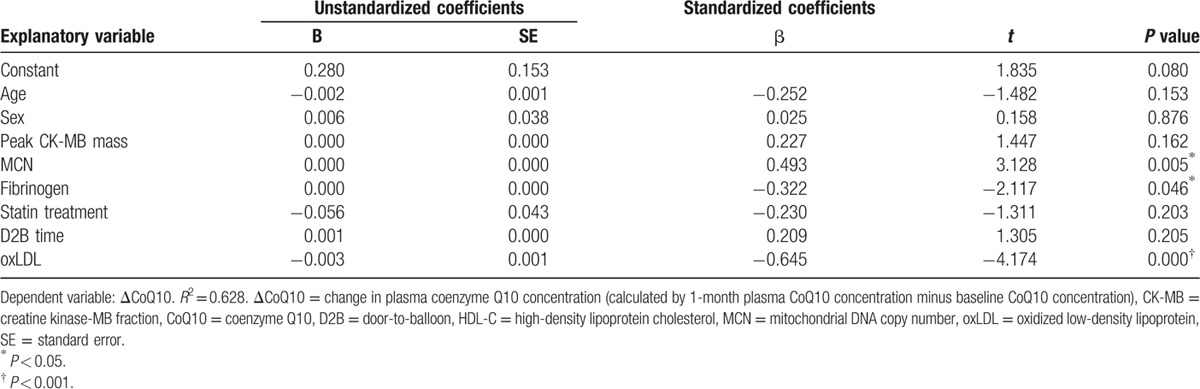
Multiple linear regression analysis of variables associated with ΔCoQ10 after primary angioplasty for acute myocardial infarction.

### 3.8 Plasma CoQ10 concentration was positively proportional to plasma ApoA1 tertile

**Figure 5:**
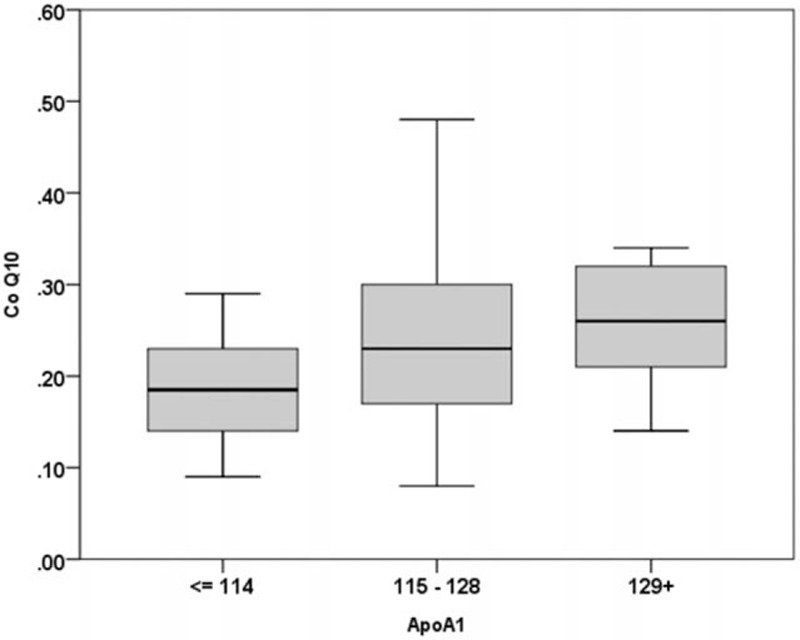
Plasma CoQ10 concentration was positively proportional to the plasma ApoA1 tertile. Patients with AMI were divided into 3 subgroups according to the baseline ApoA1 concentration tertile: Group 1, ApoA1 concentration of ≤114 mg/dL (n = 19); Group 2, CoQ10 concentration of 115 to 128 mg/dL (n = 20); and Group 3, CoQ10 concentration of ≥129 mg/dL (n = 16). Trend analysis showed that the baseline plasma CoQ10 concentration was positively proportional to the baseline plasma ApoA1 concentration (Jonckheere–Terpstra test, *P* = 0.017). AMI = acute myocardial infarction, ApoA1 = apolipoprotein A1, CoQ10 = coenzyme Q10.

Patients with STEMI were divided into 3 subgroups according to the baseline plasma ApoA1 concentration tertile. Trend analysis showed that the plasma CoQ10 concentration was positively proportional to the plasma ApoA1 concentration (Jonckheere–Terpstra test, *P* = 0.017) (Fig. 5).

## 4 Discussion

The main finding in this study is that maintenance of high endogenous levels of CoQ10 in plasma promotes recovery of LV function after STEMI. To the best of our knowledge, this is the first study to show this finding in human subjects. In the present study, the 6-month LVEF was positively proportional to the 1-month plasma CoQ10 concentration (Fig. 2). Patients with higher plasma CoQ10 concentrations at 1 month after AMI had favorable LV remodeling; specifically, they exhibited fewer changes in LVESV and greater preservation of systolic function (Figs. 3 and 4). In the multivariate linear regression model, we found that the ΔCoQ10 at 1 month was a significant predictor of LVEF 6 months after STEMI. These findings suggest that retention of CoQ10 in plasma at 1 month predicts better LV performance 6 months after primary angioplasty for STEMI. In our study, we found that the baseline oxLDL and fibrinogen concentrations were significant predictors of reduced levels of CoQ10 in plasma at 1 month. Plasma oxLDL concentration may be indicative of the body's oxidative stress burden, whereas the fibrinogen concentration may indicate inflammatory status. Our results support the argument that the cardioprotective effect of CoQ10 under conditions of ischemia–reperfusion is related to its ability to minimize AMI-related increases in oxidative stress and inflammation.^[23]^

We also found that the ApoA1 concentration was positively proportional to the baseline plasma CoQ10 concentration. In an experimental murine model of myocardial infarction,^[24]^ Dadabayev et al found that ligation of the left anterior descending coronary artery resulted in a significantly larger infarct size in ApoA1-null (ApoA1−/−) mice than in ApoA1 heterozygous (ApoA1+/−) mice, and that the pathology was traced to a functional defect in mitochondrial electron transport that arose from a deficiency in the CoQ10 pool of ApoA1-null mice. The finding suggested that CoQ10 plays an important role in mitochondrial function in cardiac tissue. This defect was reversed by intraperitoneal injection of CoQ10, which normalized the infarct size in ApoA1-null mice. The authors postulated that ApoA1 regulated CoQ10 absorption by an unknown mechanism. In the present study, we showed that the plasma CoQ10 concentration is positively proportional to the plasma ApoA1 concentration and that the MCN in leukocytes was positively correlated with ΔCoQ10. This means that patients with a higher baseline MCN have greater retention of CoQ10 in plasma 1 month after STEMI. In a murine model of myocardial infarction, Ide et al found a marked decrease in MCN in heart tissue after injury.^[13]^ Mitochondrial transcription factor A (TFAM) is essential for the maintenance of MCN. Ikeuchi et al created transgenic mice that overexpress the human TFAM gene and found that overexpression of TFAM protected against MCN truncation in heart tissue and inhibited LV remodeling after myocardial infarction.^[25]^ Mitochondria-encoded gene expression is largely regulated by the MCN.^[14]^ A decrease in the MCN results in a corresponding decrease in mtRNA and proteins, and hence mitochondrial dysfunction.^[26]^ Therefore, maintenance of a higher MCN is important to preserve mitochondrial function. However, we calculated the MCN in peripheral blood leukocytes rather in heart tissue because they are easier to obtain. In addition, studies have shown that low MCN in peripheral blood leukocytes correlates well with mitochondrial dysfunction in skeletal muscle.^[27,28]^ It is, therefore, reasonable to assume that MCN in peripheral blood leukocytes can serve as an alternative marker of cardiac mitochondrial function.

Our study may serve as a baseline for future interventional studies to examine whether CoQ10 supplementation has a beneficial effect on recovery of LV function after STEMI. Patients with STEMI who exhibit low follow-up plasma CoQ10 concentrations in the subacute stage should receive aggressive therapeutic intervention to improve long-term clinical outcomes. Elevation of the plasma CoQ10 concentration, by either lifestyle modification or pharmaceutical intervention, may provide a preventive strategy. The major limitation of this study is that it does not provide mechanistic insight. Other limitations include the relatively small size of the cohort and the fact that the MCN was measured only at baseline. Therefore, larger studies with longer follow-up durations are needed to confirm our findings.

## Acknowledgment

We would like to thank Dr Yu-Jun Chang for her contributions to the statistical analysis.
